# Scientific Software Development Is Not an Oxymoron

**DOI:** 10.1371/journal.pcbi.0020087

**Published:** 2006-09-08

**Authors:** Susan M Baxter, Steven W Day, Jacquelyn S Fetrow, Stephanie J Reisinger

**Affiliations:** National Center for Biotechnology Information, United States of America

“Many scientists and engineers spend much of their lives writing, debugging, and maintaining software, but only a handful have ever been taught how to do this effectively: after a couple of introductory courses, they are left to rediscover (or reinvent) the rest of programming on their own. The result? Most spend far too much time wrestling with software, instead of doing research, but have no idea how reliable or efficient their programs are.” —Greg Wilson [1]

As Greg Wilson's *American Scientist* article [2] circulated on the “bio-IT” e-mail lists and blogosphere this past winter, many of us sighed, groaned, and smiled in recognition. The field of computational biology crosses the span between engineering and science—a surprisingly (to some) large gulf that typically is uncovered in the process of developing scientific software.

Why opine on best practices for scientific software projects now? Computational biologists are taking on increasingly important roles in this Internet-enabled, information-rich, high-throughput era of biology [3]. Analytics and algorithms must operate on disparate and relatively large datasets. Curation and peer review is essential to critical analysis of computational conclusions. Software applications are needed to aggregate, integrate, and manage data, tools, results, and discoveries. Computational biologists are involved as advisors to technical teams developing and maintaining long-lived data resources, as product owners for software development, as coding and algorithm experts, and as reviewers of proposals and manuscripts. Whether code is developed for use in a single laboratory or as part of a larger, multi-institutional project, there are best practices worth knowing and following.

We are starting with the premise that scientific software development brings together different cultures. A “certified technology stack” might mean a robust *n*-tiered architecture to some and an expensive waste of resources to others. We want to avoid fanning controversy over interdisciplinary science [4,5] and misunderstandings inherent at the interface between engineering and science [6,7]. We hope to provide a common understanding so that we all—specialists and generalists—can work effectively on scientific software projects, increasing project efficiency, software longevity, user community acceptance, and translational impact.

We see important similarities between the way scientists and software engineers approach and attack problems which may provide a general framework for successful scientific software development. Scientists are taught the scientific method from the time they perform their first experiments. Similarly, software engineers are taught about the software development life cycle before they write their first “if” statement. By understanding similarities between these approaches, we can layer some practical methods from the software development life cycle onto computational biology projects to build a solid foundation for success.

Two of us are card-carrying software engineers; two of us are formally trained as scientists. We are all battle-scarred veterans of large scientific software development projects, while working in business, nonprofit, government, and academic settings. Many of those projects were successful; some were not. We think that the best practices learned and employed on large scientific software projects can also instruct smaller development projects carried out by single-investigator laboratories or small teams. (In addition to the references cited, see Box 1 for a suggested library and for resources to improve scientific software development processes.)

We define success as delivering a code base that produces consistent, reproducible results, is usable and useful, can be easily maintained and updated, and has a reasonable shelf life. We will also add that successful scientific software projects are usually fun—realizing this might expose how truly geeky we are.

## Suggested Best Practices

### 

To achieve success in scientific software projects, we propose a minimal set of guidelines for pragmatic practitioners, peer reviewers, and project leaders of small- (single-lab) to medium- (collaborative, noncommercial projects) sized projects. We debated, solicited advice, reread some of our favorite books [8,9], and took guidance from our editors, to boil down our experiences and this enormous topic to five recommended, stripped-down practices for successful scientific software development: 1) design the project up-front; 2) document programs and key processes; 3) apply quality control; 4) use data standards where possible; and 5) incorporate project management. We can trace project failures back to breakdowns in any one or more of these practices. We will next explain what we mean by each practice.

#### Design the project up-front.

Good scientists do not perform experiments before developing a hypothesis, then describing materials and methods to test that hypothesis. Similarly, before the first line of code is written, software projects should be proactively and thoughtfully designed. This does not necessarily require a voluminous tome, but it should answer two key questions: “What will the program(s) do?” and “How will the results produced by the program be verified?” The most simple design documents describe inputs, how those inputs will be transformed by the program(s), and outputs.

**Box 1. Suggested Resources and Beginning Library for Maturing Scientific Software Developers and Project Managers**

**Broad scope:**

Basic software development practices: Software carpentry. http://www.scipy.org. *Website and coursework written with scientific software development in mind*.

**Design:**

Gamma E, Helm R, Johnson R, Vlissides J (1995) Design patterns: Elements of reusable object-oriented software. Boston: Addison-Wesley. 395 p. *Seminal book on object-oriented design patterns for developers aiming to reuse code bases.*

**Quality Control:**

Lewis WE (2004) Software testing and continuous quality improvement. 2nd edition. New York: Auerbach. 560 p. *Squarely addresses testing and quality in a maintenance environment; of particular use to developers supporting long-lived code bases.*

**Project management:**

Berkun S (2005) The art of project management. Sebastopol (California): O'Reilly Media. 488 p. *Easy-to-read book filled with many “lessons learned,” to read rather than to have to experience.*

Schwaber K (2004) Agile project management with Scrum. Redmond (Washington): Microsoft Press. 192 p. *Iterative methodology aimed at keeping plans in sync with what is really going on in software development projects.*

Based on the purpose of the software, identifying the appropriate technologies or programming languages is a vital decision during the design phase. While typically driven, often mistakenly, by the current in-house expertise of the software developers, there should be careful analysis in addressing the problem with the most practical selection of technologies. For example, if ease of distribution is considered important, the platform-independent nature of Java may make the most sense; if the software deals with a great deal of text manipulation, Perl may be best suited; if speed of execution is essential, C or C++ may be the way to go. In addition to considering the built-in strengths of a particular language, most offer a vast array of canned libraries (whether included in the distribution or preexisting as an open source project) developed to handle all but the most arcane technological issues. It is at this design juncture that much time can be saved in building software components that could be acquired for almost nothing through relatively minimal research. Additionally, plugging in trusted, reusable code bases lends credibility to the overall quality of the software and streamlines the testing phase.

The team should develop test plans and create data to test their code. In the development of test plans, it is also good practice to consider independent variables, such as how long the program might take to run on a certain platform, how it will work with a real-world-sized input file, or how well it will interface or interoperate with other programs that are not a part of your project.

The design phase should also address software usability requirements. If the software under development will be used only by the programmer, usability might not be a large concern. However, as funding agencies emphasize dissemination, collaborative teams aim to share tools; and to use statistics to help justify renewal of funding, usability should be a higher priority in scientific software development. Designing facile user interfaces, interactive feedback cycles, maintenance and release plans, or easy reuse of code or tools requires careful thought, due diligence, and resourcing up-front.

Typically, the proposal writing or management approval process provides a mechanism to force project design. Before coding begins, projects can discover existing tools and data standards and articulate the planned functionality and testing of the software. No matter the scale of the software project, it is important to incorporate feedback from key stakeholders (thesis advisor, external advisory committee, etc.) in this process to ensure that the design meets expectations.

#### Document programs and key processes.

One of the foundations of scientific research is the lab notebook, where materials, methods, and results are recorded so that experiments can be repeated. Similarly, all computer programs and code bases should be well-documented, modular, and easy to read and follow even by users who did not write the program. Modularity can be a complex issue, but at a basic level it refers to coding in a way so that the overall task being performed is divided into small, discrete units of work. This design paradigm promotes reusability and flexibility [10]. A modest level of documentation might provide help through a user-guide, information on how to compile and execute a program, and in-line comments describing program functions and modules.

#### Use a quality control process.

One cornerstone of good science is reproducibility of results. Similarly, being able to consistently reproduce the results of a computer program is the yardstick used to measure the validity of that program. Reproducibility requires three things: ensuring a program works the way it should (testing), knowing exactly what was used to produce the results (version control), and recognizing and tracking program bugs.

Programs should be thoroughly tested according to the test plans developed in the design phase. Well-designed unit tests may be used to address whether a particular module of code is working properly and allows testing to proceed piecemeal and iteratively throughout the development process. This enables bugs to be identified and handled early so as to avoid major problems during integration and final testing.

Undeniably, computational biology projects are fluid: there are always newer, better data files and standards available, requiring continual updates to the code base. Consequently, it is critical to track exactly which version of software and which set of input files and parameters were used to produce a specific set of results. This is especially important six months later, when the original programmer has moved on to another project. Developers should use version control for both data and source code, tying results to specific versions. Subversion [11] and CVS [12,13] are open source version control systems freely available. Finally, confessing to and tracking known bugs should be encouraged since bugs are to be expected in software products. Jira [14] and Bugzilla [15] are widely used issue-tracking tools.

Beyond application of functional testing, quality can be addressed further through performance optimization using bounds checkers (e.g., Valgrind provides basic debugging capabilities plus detailed profiling of memory use). These issues are typically overlooked during software development as problems with memory leaks and poor memory management hide behind software functionality and may long go unnoticed.

#### Apply data standards where possible.

Disseminating to and sharing results with the broader research community is critical and often provides the basis for new scientific progress. The same is true for computer programs. The inputs, outputs, and “results” of computer programs are often data files. Whether included as supplementary materials for a manuscript or as subtables in an enterprise level relational database, scientific data should be supplied in accepted, standard formats wherever possible. Admittedly, biology is a fast-moving target. However, the increasing need to share, compare, and integrate data and tools is driving communitywide initiatives to standardize biological data formats [16,17]. As one example, the MGED Society has defined minimal sets of parameters to describe gene expression array datasets (MIAME), along with a data standard (MAGE) [18]. As a result of their lead and other work, shared repositories for microarray results are now available and evolving [19,20], and journals are increasingly requiring supplemental data deposition at them [21]. Software developers should research the availability of community-accepted data standards as inputs and outputs for their programs. Even if suitable data standards are not available, it is important to include documentation (metadata) describing the data. Metadata should explain the format (syntax) of the data as well as definitions and assumptions that allow the data to be interpreted or used in the proper context (semantics). Data standards ensure the ability to scale and integrate code bases, enable accurate and efficient code development, and reduce user and peer reviewer frustration.

#### Incorporate project management.

In scientific research, principal investigators ensure that experiments are performed according to defined procedures, while making progress in the context of a schedule and a budget. For software development projects, a project manager performs a similar function. Principal investigators who are not themselves software engineers may find themselves filling a project manager role because they supervise people in their labs who write software. Project management for a modest algorithm-development project involving one or two programmers might involve informal design and code reviews, regular meetings to track progress against an established timeline, and review (and sign-off) of testing results.

Larger, collaborative projects, however, can become hopelessly chaotic without more disciplined project management. A commonly used approach to managing larger projects is to break them into manageable subprojects, with a series of release cycles interleaved with user or stakeholder feedback. A simple project website, wiki, or more sophisticated solutions such as Xplanner [22] and Basecamp [23], can be used to facilitate team communication, share project plans and documentation, and transparently manage development projects. In our opinion, the Scrum software development methodology [24] offers a practical way to iteratively manage medium-sized software projects.

## Examples of Successful Projects That Employ Best Practices

Outside our own anecdotal experiences, we think there is growing evidence that software best practices can effectively meet real-life, scientific needs. We can point to heavyweight projects, such as the cancer Biomedical Informatics Grid (caBIG) [25], and to more modest, lightweight activities such as the Bioconductor project [26].

Specifically, the Bioconductor project has adopted practical techniques that are instructive for small software projects [26]. The Bioconductor project develops statistical software packages ubiquitously employed in biomedical research. This group recognized that reproducing computational research reported in the literature is usually hampered by poorly documented software packages. While scientific manuscripts now typically point to supplementary materials (usually data and computer programs) on the Internet, access to them is not always enough to replicate the research reported. This open-source project adopted the concept of a “vignette,” which is a detailed and interactive document providing a textual description of software functionality [27]. This form of documentation, long regarded as a software best practice, has engendered quite a cult following in the scientific community. In this case, the ultimate goal of reproducible research has exposed software best practices as an *enabler* and not as a burdensome side effect.

Reading back over this article, we recognize that there are many “shoulds” in our guidelines. In our defense, we write from our collective, heartbreaking experiences watching wheels reinvented, finding dead or unusable programs, and, worse, inheriting rancid and labyrinthine code bases. We are of the opinion that community adherence to the guidelines described here will increase the impact and usability of computational biology work, without placing undue burden on the creators of rapidly evolving, scientific code bases.
